# Synthesizing Carbon Quantum Dots via Hydrothermal Reaction to Produce Efficient Antibacterial and Antibiofilm Nanomaterials

**DOI:** 10.3390/foods13010058

**Published:** 2023-12-22

**Authors:** Tianqi Cui, Ya Fan, Yaping Liu, Yangyue Ding, Xinyue Li, Guiguang Cheng, Jianjun Cheng

**Affiliations:** 1 Faculty of Food Science and Engineering, Kunming University of Science and Technology, Kunming 650550, China; 2 College of Food Science, Northeast Agricultural University, Harbin 150030, China

**Keywords:** carbon quantum dots, antibacterial activity, antibiofilm, membrane-damage, resistance

## Abstract

This study aimed to synthesize antibacterial carbon quantum dots (SP-CDs) from polyethyleneimine and spermidine via hydrothermal reaction. It was revealed that SP-CDs, with small size (7.18 nm) and high positive charge (+31.15 mV), had good fluorescence properties and lots of amino groups on their surfaces. The inhibition effect of SP-CDs on *Staphylococcus aureus* was better than that towards *Escherichia coli*, and the SP-CDs also had an inhibitory effect on multi-drug-resistant *E. coli*. The mechanism of SP-CDs shows that the SP-CDs were adsorbed on the surface of the negatively charged cell membrane through electrostatic interaction. SP-CDs can cause changes in membrane permeability, resulting in a shift of the cell membrane from order to disorder and the decomposition of chemical components, followed by the leakage of cell contents, resulting in bacterial death. SP-CDs can also significantly inhibit biofilm formation, destroy mature biofilms and reduce the number of living cells. Moreover, SP-CDs had negligible antimicrobial resistance even after 18 generations of treatment. This study proves that SP-CDs effectively inhibit the proliferation of foodborne pathogens, providing new feasibility for the application of carbon-based nanomaterials in the food industry.

## 1. Introduction

Foodborne diseases caused by foodborne pathogens occupy a high proportion of food safety incidents and are one of the major public health problems worldwide. *Escherichia coli* and *Staphylococcus aureus* are typical Gram-negative (G^−^) and Gram-positive (G^+^) pathogens. Most *E. coli* are symbiotic bacteria, and some can even be used as probiotics, such as *E. coli* Nissle 1917 (EcN), a unique endotoxin-free, biosafe and non-pathogenic strain that has received widespread attention for its probiotic function and ability to prevent gastrointestinal diseases [1,2]. However, there are also strains that are opportunistic or highly pathogenic to humans when consumed through food (e.g., strains belonging to serotype O157:H7, etc.) [3,4]. *S. aureus* is widely found in the environment and secretes virulence factors that can easily contaminate meat, egg products and milk [5]. Additionally, *S. aureus* can produce enterotoxins in food, which can cause food-borne poisoning [6]. In addition, both bacteria can attach to the surface of food and food processing equipment to form biofilms [7]. Microbial contamination by bacterial biofilms is a serious threat to public health [8]. In addition, incomplete elimination of bacterial biofilms can easily lead to persistent microbial contamination and superbugs [9]. Conventional antibiotic treatment often leads to antibiotic resistance in bacteria, making the suppression of foodborne pathogens more challenging. Therefore, it is urgent to develop new antibacterial and biofilm inhibitors that can avoid bacterial resistance.

Antibacterial nanomaterials have attracted much attention due to their unique antibacterial activity and different antibacterial mechanism from traditional antibiotics [10]. The antimicrobial mechanisms of some nanoparticles can be attributed to respiratory chain damage, lipid peroxidation, inactivation of intracellular proteins and disintegration of bacterial cell membranes [11,12]. Due to the complex antibacterial mechanism of nanoparticles, the development of antibiotic-resistant bacterial strains can be avoided [13]. In recent years, carbon-based nanomaterials, such as carbon nanotubes, carbon nanofibers, carbon nanospheres and carbon quantum dots (CDs) have been shown to have effective antibacterial efficacy [14]. However, there are still some health problems with certain metal nanoparticles. CDs have many advantages such as simple synthesis method, low cost, good dispersion and controllable surface chemical structure [15]. Due to their low toxicity and high biocompatibility, CDs have been used in the food field as an antibacterial material in smart food packaging. 

CDs can be synthesized via microwave, hydrothermal, ultrasonic and electrochemical methods, among which the hydrothermal method is the most convenient and efficient [16]. When CDs are prepared via the hydrothermal method, different raw materials can provide different functional groups on the surface of the CDs, and these physical and chemical properties endow CDs with antibacterial activity. Polyethylenimine is a kind of hydrophilic polymer with antibacterial activity. Demirci et al. prepared nitrogen-doped CDs using polyethylenimine and citric acid as precursors via a hydrothermal method. The synthetic CDs had no obvious toxicity to mammalian cells and can inhibit the growth of both G^−^ and G^+^ [17]. Spermidine is a natural polyamine that binds and stabilizes DNA and RNA and is essential for cell growth, proliferation and tissue regeneration [18,19]. The fluorescent carbon quantum dots (SPD-CQDs) synthesized by Li et al. using spermidine in two steps can reduce the minimum inhibitory concentration (MIC) of spermidine by 25,000 times. In addition, SPD-CQDs showed antibacterial activity against a variety of bacteria such as *E. coli* and *S. aureus* as well as multi-drug-resistant bacteria [20]. This provides the possibility of preparing polyethylenimine and spermidine carbon dots with antibacterial and the ability to destroy biofilms.

In this study, spermidine and polyethylenimine were used to synthesize carbon quantum dots (SP-CDs) via a one-step hydrothermal reaction. The SP-CDs were characterized using a variety of techniques to clarify their structure. The antibacterial and anti-biofilm activity of SP-CDs against *S. aureus*, *E. coli* and multi-drug-resistant *E. coli* were explored and further clarify their antibacterial mechanism. In addition, the microbial resistance of SP-CDs was further analyzed. It is hoped that SP-CDs can be used as an alternative method to obtain efficient, low-cost and environmentally friendly antimicrobials and eventually achieve the eradication of bacterial biofilms from the food industry.

## 2. Materials and Methods

### 2.1. Materials

The polyethyleneimine (PEI, 99%, molecular weight 10,000) was obtained from Macrlin Biochemical Technology Co., Ltd. (Shanghai, China). The spermidine (98%) was purchased from Yuanye Biotechnology Co., Ltd. (Shanghai, China) Cat. no. L7012 Live/Dead BacLight Bacterial Viability Kit for microscopy was obtained from Thermo Fisher Scientific (Waltham, MA, USA). The Luria Bertani (LB) medium was purchased from Obo Star Biotechnology Co., Ltd. (Beijing, China).

### 2.2. Strain and Bacteria Culture

The *S. aureus*, *E. coli* and multi-drug resistance *E. coli* (MREC) were presented by Prof. Fengling Bai in Bohai University. The *S. aureus*, *E. coli* and MREC were incubated in LB broth at 37 °C for 24 h in passaging. The obtained bacterial suspensions were centrifuged and the bacteria thallus was washed twice with phosphate-buffered saline (PBS, pH = 7.2, 0.2 mol/L) and resuspended in 0.9% saline. The optical value of UV–vis at 600 nm of the final bacterial suspensions was adjusted to 0.1, then placed at 4 °C for use.

### 2.3. Synthesis of SP-CDs

**Figure 1:**
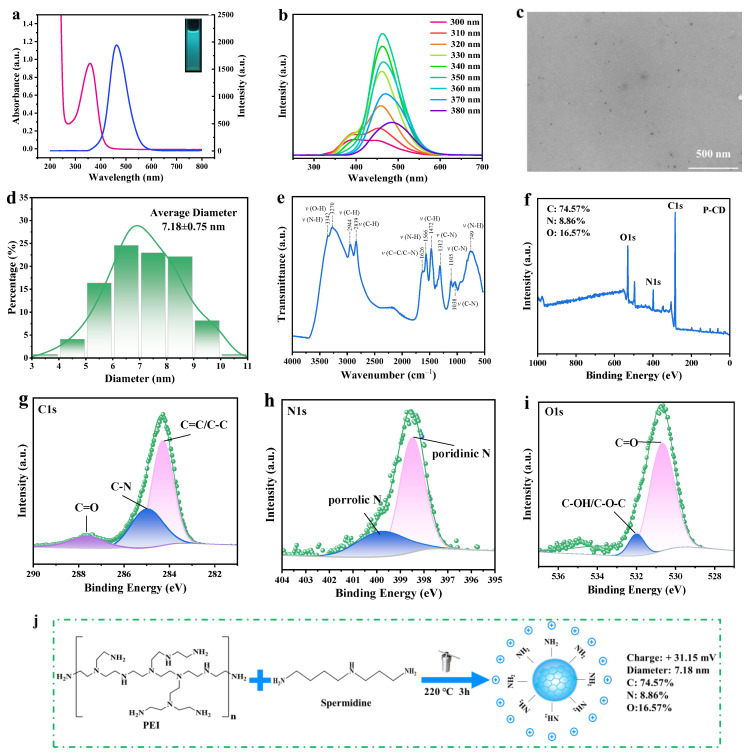
Characterization of SP-CDs. (**a**) UV–vis absorption spectra, the illustration shows the fluorescence emission of SP-CDs under a 365 nm violet lamp. (**b**) Excitation (Ex) and emission (Em) spectra, (**c**) TEM images, (**d**) particle size distribution, (**e**) FT-IR spectra, (**f**) XPS wide sweep and high resolution (**g**) C1s, (**h**) N1s and (**i**) O1s spectra. (**j**) One step hydrothermal reaction synthesized SP-CDs.

The hydrothermal synthesis method was used to synthesize SP-CDs according to Cui et al. with some modified [21]. The synthesis route of the SP-CDs is shown in Figure 1j. The SP-CDs were synthesized by adding 2.5 g of PEI and 2.5 g of spermidine to a polytetrafluoroethylene-lined hydrothermal autoclave containing 250 mL of deionized water, which was fully dissolved and then placed in an oven and heated at 220 °C for 3 h. After the SP-CDs suspension was cooled to room temperature, it was filtered through a 0.22 μm filter. The SP-CDs were freeze-dried under vacuum for use.

### 2.4. Characterization of SP-CDs

An H-700 electron microscope operating (Hitachi, Tokyo, Japan) at 200 kV was used to obtain the transmission electron microscopy (TEM) images of the SP-CDs. A K-Alpha X-ray photoelectron spectra (XPS) system (Thermo Fisher Scientific, Waltham, MA, USA) was used to collect the XPS data of SP-CDs for the analysis of the surface chemical elements. A UV–vis spectrophotometer (T9s, Beijing, China) was used to record the UV–vis absorption spectra of the SP-CDs, and the scanning range was 200–800 nm. A fluorescence spectrophotometer (F-7000, Hitachi, Japan) was used to measure the excitation and emission spectra of the SP-CDs. An FT–IR spectrometer (Nicolet iS10, Thermo Fisher, USA) was used to identify the chemical functional groups of SP-CDs. The FT–IR spectra were recorded in a range of 4000–500 cm^−1^ at 4 cm^−1^ resolutions. 

### 2.5. In Vitro Antibacterial Activity of SP-CDs

#### 2.5.1. Minimum Inhibitory Concentration (MIC)

The determination of the MIC of the SP-CDs was performed using a 96-well microplate double dilution method [22]. The SP-CD powder was dissolved in LB broth to prepare an SP-CDs solution of 1024 μg/mL. First, 200 μL of SP-CDs was added to well #1, and LB liquid culture medium was added to well 2–10. Then, 100 μL of solution from well 1 was added to well #2; similarly, 100 μL of solution was added from well #3 to #10. The control group used LB broth instead of SP-CD solution. Approximately 10^6^ CFU/mL of bacterial suspension was added to each well and incubated in a 37 °C incubator shaker at 110 rpm for 24 h. 

#### 2.5.2. Determination of Bacteriostatic Circle

The Oxford cup punching method was used to determine the antibacterial activity of the SP-CDs. The Oxford cups were placed in a sterile petri dish and then poured into a uniform mixture of 25 mL LB medium and 10^6^ CFU/mL suspension (1 mL) of bacteria. Each Oxford cup was removed after the medium solidified, and 180 μL of SP-CDs with 1, 2 and 4 MIC were added to the well, respectively. All the petri dishes were cultured at 37 °C for 24 h. According to the measured bacteriostatic circle, the bacteriostasis rate was calculated [23]:

Bacteriostasis rate (%) = (*D_sample_* − *D_control_*)/*D_sample_* × 100%

where *D_sample_* and *D_control_* are the diameter of the bacteriostatic circle produced via SP-CD treatment and in the control group.

#### 2.5.3. Flat Coating Method

SP-CDs of 1/4 MIC, 1/2 MIC and 1 MIC were added to a bacterial suspension of 1 × 10^6^ CFU mL^−1^. The bacteria and SP-CDs cultures were incubated and diluted 10 times with 9% sterile normal saline. The control group was not treated with SP-CDs. The diluted co-cultures (100 µL) were evenly spread on LB agar plates. The number of colonies on each agar plate was counted to calculate the antibacterial activity [24]. 

### 2.6. Antibacterial Mechanism of SP-CDs

#### 2.6.1. Bacterial Surface Charge Measurement

The surface charges of *S. aureus*, *E. coli* and MREC were determined according to Ramalingam et al., 2016 [25]. Centrifugation at 10,400× *g* was performed for 10 min after treating the bacterial suspensions with 1 MIC SP-CDs for 24 h. The bacteria cells were washed twice with sterile PBS and resuspended in PBS. The Zeta potential values of the bacterial cells were measured using a Zetasizer Nano-ZS90 (Malvern Instruments, Malvern, UK).

#### 2.6.2. FT-IR Analysis of Bacterial 

The surface functional groups of the three bacterial strains were determined using FT-IR according to Wang et al. 2022 [5]. The suspensions were treated with 1 MIC SP-CDs and cultured at 37 °C for 24 h, with untreated bacteria as the control. The bacterial cells were obtained via centrifugation at 10,400× *g* for 10 min and washed twice with sterile PBS. After gradient dehydration, we ground the cells in a mortar with KBr (1:100), and the FT–IR spectra of the bacteria were recorded at 4000–500 cm^−1^ using a FT–IR spectrometer (Nicolet iS10, Thermo Fisher, Waltham, MA, USA). 

#### 2.6.3. Confocal Laser Scanning Microscope (CLSM) Observation

The cultures of bacterial suspensions (1 × 10^8^ CFU mL^−1^) and SP-CDs were centrifuged at 10,000× *g* for 10 min to obtain the bacteria cells.

The co-culture of SP-CDs and 1 × 10^8^ CFU mL^−1^ of bacterial suspension was centrifuged at 10,000× *g* for 10 min to obtain the bacterial cells, washed twice with 8.5% normal saline and then re-suspended in 1 mL of the same solution, to which 3 µL SYTO 9 and PI were added and incubated in the dark for 15 min. A confocal laser scanning microscope (Leica TCS SP5, Leica Microsystems GMBH, Heidelberg, Germany) was used to observe the fluorescence images.

#### 2.6.4. Scanning Electron Microscope (SEM) Observation

The bacterial suspension was added with 1 MIC SP-CDs and cultured at 37 °C for 12 h. The precipitate was collected by centrifuging (500× *g*, 15 min) and resuspended in PBS, and the bacteria cells obtained after re-centrifugation were fixed with glutaraldehyde 2.5% solution overnight. Then, they were dehydrated with ethanol solutions at the dehydration gradients of 30, 50, 70, 90 and 100%, respectively, with each gradient dehydrated for 15 min. A scanning electron microscope (Hitachi, Japan) was used to observe the morphology and surface characteristics of the strains after sputter-coating with gold. 

#### 2.6.5. Transmission Electron Microscope (TEM) Observation

The bacterial suspensions treated with SP-CDs were diluted to an appropriate concentration and adsorbed using a special copper mesh for TEM. An H-700 TEM (Hitachi, Japan) was utilized to observe the structure of the adequately dried bacteria.

#### 2.6.6. Relative Amount of Extracellular Nucleic Acids of Bacteria

The method according to Wang et al. [26] was followed. First, 20 mL of prepared bacterial suspension and 1 MIC of SP-CDs were co-cultured at 37 °C for 12 h. The bacterial suspensions were centrifuged at 1000× *g* for 15 min every 2 h to collect the supernatant and the absorbance was measured at 260 nm using a UV–vis spectrophotometer (T9s, Beijing, China). 

#### 2.6.7. Relative Number of Extracellular Proteins of Bacteria

The effects of SP-CDs on soluble proteins of bacteria were determined with the BCA kit. The bacterial suspensions of 10^6^ CFU/mL were added to LB broth containing 1 MIC SP-CDs and incubated for 12 h at 37 °C, followed by centrifugation at 1000× *g* for 15 min every 2 h. The protein content was measured using the BCA kit and a curve was drawn based on the change in protein content measured at different times.

### 2.7. Antibiofilm Activity

Firstly, the antibiofilm activity of SP-CDs was determined by crystal violet staining [27]. The bacterial suspensions were treated with 0 MIC, 1/4 MIC, 1/2 MIC and 1 MIC of SP-CDs. The supernatant was removed after incubation at 30 °C for 24 h and rinsed with PBS for 3 times, and the bacterial cells were stained with 0.4% crystal violet for 5 min. After rinsing with sterile water and drying for 10 min, to each well was added 200 μL 95% ethanol, and the OD_570_ was measured using an enzyme-labeled meter. Each sample was repeated three times.

Secondly, the number of bacteria was measured using the spread plate method [28]. Firstly, 2 mL LB broth and 20 μL bacteria suspension of 1 × 10^6^ CFU/mL were added to 24-well plates (Corning, NY, USA) with an aseptic coverslip at the bottom. Then, SP-CDs with 1/4 MIC, 1/2 MIC and 1 MIC were treated at 37 °C for 24 h, respectively. The coverslip was cleaned with PBS 3 times and then placed in LB broth for 2 min of vortexing to completely release the bacterial cells. The bacterial suspension was serially diluted 10-fold and 100 μL was spread evenly on LB agar. The total number of bacterial colonies was counted.

Finally, biofilm formation was observed via CLSM [29]. According to the above method, the bacteria growth on the coverslip at 37 °C was given 48 h to form biofilm. The biofilm formation was then detected by staining in the dark for 15 min with the L7012 LIVE/DEAD bacterial staining kit. A confocal laser scanning microscope (Leica TCS SP5, Leica Microsystems GMBH, Heidelberg, Germany) was used to observe the fluorescence images. 

### 2.8. Microbial Resistance Assay

The possibility of SP-CDs to have an antimicrobial resistance effect to bacteria was detected with reference to Yang et al. [30]. In detail, 20 μL of *S. aureus*, *E. coli* and MREC cells were transferred into 2 mL of sterile LB broth containing 1/2 MIC of SP-CDs. After culturing at 37 °C for 24 h, the bacterial cell proliferation was calculated by detecting the absorbance. If the bacteria cells adapted to a concentration of 1/2 MIC and the cell proliferation was >85%, the treatment concentration of SP-CDs became 1 MIC. Otherwise, the SP-CDs continued to treat the bacteria at a concentration of 1/2 MIC and were incubated for 24 h. The concentration of the SP-CDs was gradually increased with a concentration gradient of 1, 2, 4, 8, 16, 32 and 64 MICs, and the above treatment process was repeated at 24 h intervals. We recorded the sub-MIC for each day of the 18-day period.

### 2.9. Statistical Analysis

All experiments and their associated statistical analyses were conducted in triplicate and the results were expressed as mean ± standard deviation (SD). A significance level of 0.05 was used. The statistical analyses were performed using SPSS 19.0 (IBM SPSS Statistics, Armonk, NY, USA) software, and the drawings were performed using Origin Pro 2020 (Origin Lab Corporation, Northampton, MA, USA). 

## 3. Results and Discussion

### 3.1. Performance Characterization of SP-CDs 

#### 3.1.1. Optical Properties of SP-CDs

SP-CDs can show bright blue fluorescence under a 365 nm violet lamp (Figure 1a); this photoluminescence feature is one of the most important characteristics of CDs and is also one of the signs of the formation of CDs. The UV-vis absorption spectra of SP-CDs showed a wide absorption peak near the wavelength of 350 nm, which is mainly attributed to the N−π* transition caused by the C−N or C=O bond in the chemical composition (Figure 1a). This is also consistent with the obvious characteristics of carbon quantum dots, that is, they have a strong absorption peak in the ultraviolet region. In addition, the SP-CDs had photoluminescence and good optical stability. An excitation light with a wavelength of 300–400 nm (increment of 10 nm) was used to characterize the emission spectrum of the SP-CDs and thus reveal the fluorescence emission behavior of SP-CDs, (Figure 1b). With the increase in excitation wavelength, the fluorescence emission peak of the SP-CDs gradually redshifted from 446 nm to 508 nm, and the maximum fluorescence intensity was obtained at 350 nm excitation, corresponding to the emission wavelength of 462 nm. The fluorescence intensity decreased with the further increase in excitation wavelength. 

#### 3.1.2. Microstructure of SP-CDs

The SP-CDs were spherical particles with good dispersion and no obvious aggregation (Figure 1c). The particle sizes of about 122 monodisperse SP-CDs in TEM images were analyzed using software Image J 1.8.0. The results show that the particle sizes of the SP-CDs were mainly in the range of 3.65–10.09 nm (Figure 1d). In addition, according to the statistical results, the average particle size of the SP-CD_S_ was approximately 7.18 nm, which is the typical particle size range of CDs.

#### 3.1.3. Chemical Composition of SP-CDs

The FT-IR spectrum of the SP-CDs is shown in Figure 1e. There was a peak at 3342 cm^−1^, which corresponded to the stretching vibration of O−H, and the characteristic peak at 3270 cm^−1^ was attributed to the amino/amide N−H bond stretching vibration. This result suggests that SP-CDs have a large number of amino groups attached to their surfaces [31]. The vibration absorption bands located at 2944 and 2839 cm^−1^ were assigned to C−H, and the weak peak near 1629 cm^−1^ belongs to the stretching vibration of C=C or C=N bonds in the carbon core skeleton structure [32,33]. The absorption peak near 1566 cm^−1^ corresponded to the bending vibration of N−H, and the absorption peaks near 1312, 1105, and 1038 cm^−1^ were related to the stretching vibration of C−N. According to the above results, there were a large number of amide groups and N-related infrared absorption peaks on the surfaces of the SP-CD_S_, indicating that PEI and spermidine as raw materials can achieve effective N-doping.

The results of an XPS element sweep show that the signal peaks at 284.1, 398.1 and 531.1 eV of XPS binding energy were C1s, N1s and O1s, respectively (Figure 1f). The relative element content of the main compositional elements C, O and N were 74.57%, 8.86% and 16.57%, respectively. Because the N-rich precursor easily participates in the synthesis reaction, the content of N element in the SP-CDs was high. It can be seen from the C1s spectrum in Figure 1g that SP-CDs mainly have three signal peaks at 284.3 eV, 285.0 eV and 287.6 eV, and the corresponding main forms of the C element were C−C/C=C, C−N and C=O/C=N [34]. C−C/C=C is the main component of the SP-CDs skeleton, which is related to intramolecular dehydration. In addition, according to the C1s peak splitting results, the peak splitting area of XPS related to the C=O/C=N bond was small, indicating that the surface composition of SP-CDs contained only a small amount of carbonyl groups, while the peak splitting area of XPS related to the C−N bond was large, indicating that spermidine can effectively participate in the formation of CDs carbon nuclei. The N1s spectrum showed two XPS signal peaks at 398.5 eV and 399.7 eV. The main peak of 398.5 eV usually attributable to sp^2^ N is involved in triazine rings [35], while the signal peak at 399.7 eV was attributed to bridging N atoms in N-(C)_3_ or N bonded with H atoms [36]. From the O1s spectrum (Figure 1i), it can be seen that the peaks of O1s with binding energies of 530.7 and 532.0 belong to C=O, C−OH/C−O−C [37]. According to the above results, spermidine contains a lot of amine groups (−NH_3_) on its surface, and as a nitrogen dopant, it can effectively realize the nitrogen doping of CDs. Moreover, the SP-CDs had a large number of N- and O-containing functional groups on their surfaces, and the existence of these polar groups is the main reason that SP-CDs have good water solubility. SP-CDs have good water solubility and dispersibility, so they can be evenly dispersed in a food matrix. 

### 3.2. Antibacterial Activity of SP-CDs

The SP-CDs had certain antibacterial activity against *S. aureus*, *E. coli* and MREC. The SP-CDs had the best antimicrobial effect against *S. aureus* with a MIC of 64 μg/mL, and against *E. coli* with a MIC of was 128 μg/mL. The SP-CDs also had antibacterial activity against drug-resistant bacteria, while the MIC of MREC was 256 μg/mL. 

**Figure 2:**
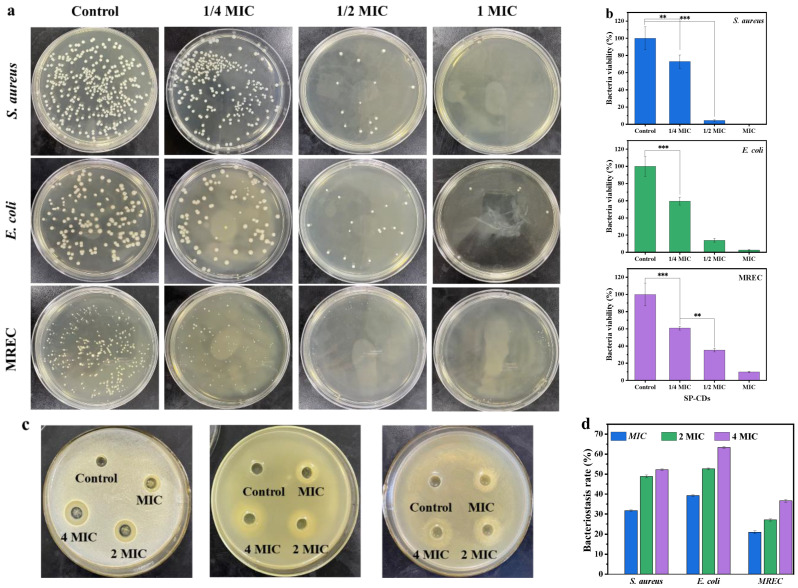
Antibacterial activity of SP-CDs. (**a**) Comparison of bactericidal activity of SP-CD treatments against *S. aureus*, *E. coli* and MREC determined by counting the number of bacterial colonies on the agar plate. (**b**) Bacterial viability of *S. aureus*, *E. coli* and MREC after treatment with SP-CDs. (**c**) The inhabitation zone diameter of *S. aureus*, *E. coli* and MREC after treatment with SP-CDs were detected via the Oxford cup punching method. (**d**) Bacteriostasis rate of SP-CDs against *S. aureus*, *E. coli* and MREC according to the results of the inhabitation zone diameter. ** *p* < 0.05, *** *p* < 0.001.

The antibacterial activity of SP-CDs against *S. aureus*, *E. coli* and MREC was investigated via the plate coating method. The total number of colonies of all three strains decreased as the concentration of SP-CDs increased (Figure 2a). When treated with the MIC of SP-CDs, there were almost no visible colonies of *S. aureus* and MREC on the solid medium, and for *E. coli*, although colony counts were present, we also found significant reduction compared to the control. The bacterial viability of *S. aureus* decreased significantly by about 27% and that of *E. coli* by about 40% when treated with 1/4 MIC of SP-CDs (Figure 2b). With the concentration of SP-CDs was increased to 1/2 MIC and the MIC, its inhibitory effect on *S. aureus* was even stronger. However, when the concentration of SP-CDs was 1/2 MIC, the bacterial viability of *S. aureus* decreased to 4% and that of *E. coli* decreased to 14%. The above results indicate that the inhibitory effect of SP-CDs on the three strains of bacteria shows dose dependence.

Secondly, the bacteriostatic effect of SP-CDs was measured via bacteriostatic zone and bacteriostatic rate (Figure 2c,d). SP-CDs of MIC, 2 MIC, and 4 MIC had an obvious inhibition zone with a diameter greater than 12 mm. The diameter of the antibacterial zone significantly increased with increasing SP-CD concentration (*p* < 0.05), indicating that the antibacterial effect on the three strains was gradually enhanced. When the concentration of SP-CDs was MIC, the antibacterial zone diameter of *S. aureus* was 11.72 mm and the antibacterial rate was 31.47%, and when the concentration was increased to 2 MIC, the antibacterial zone diameter reached 15.63 mm and the antibacterial rate also increased to 48.83%. In addition, after treatment with SP-CDs of 4 MIC, the antibacterial zone diameter of *S. aureus* was 16.74 mm and the antibacterial rate was 52.21%, while the antibacterial zone diameter of *E. coli* was 21.87 mm and the antibacterial rate was 63.42%. The antibacterial diameters of SP-CDs of MIC−4 MIC on MREC were all greater than 10 mm., and the bacteriostatic rate was 31.03%–36.61%. 

### 3.3. Antibacterial Mechanism of SP-CDs

#### 3.3.1. Neutralization of Bacterial Surface Charge

**Figure 3:**
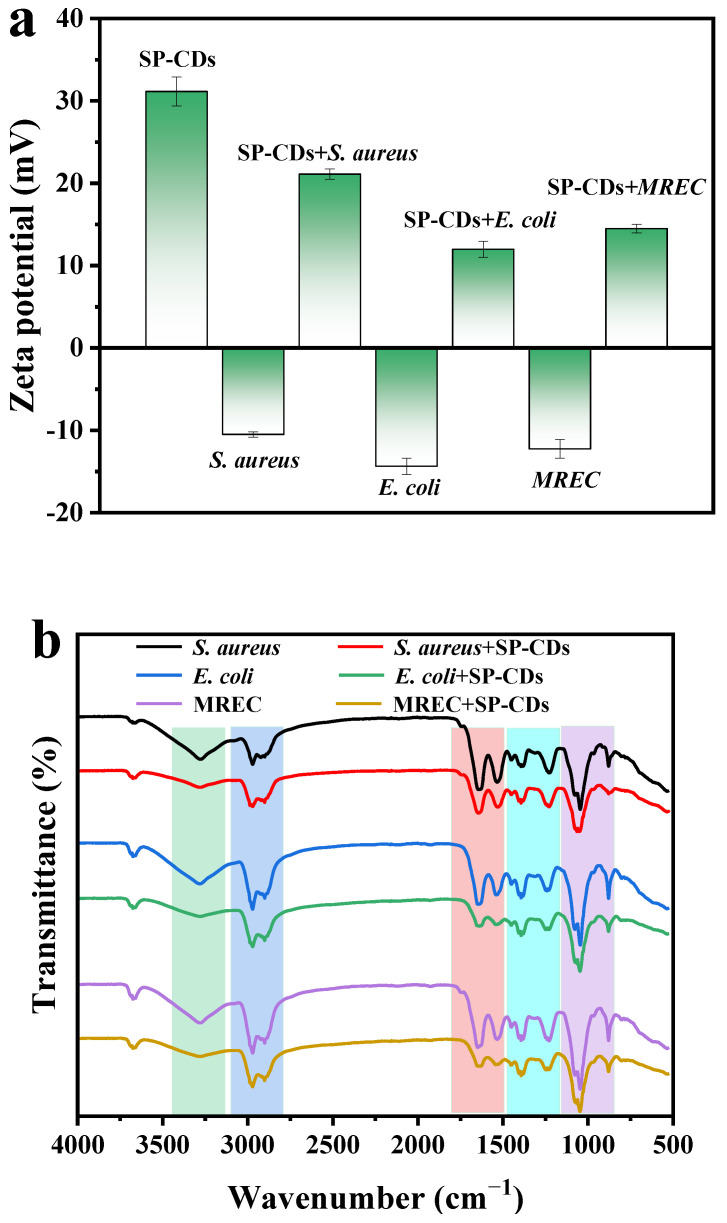
Effect of SP-CDs on bacterial cell surface. (**a**) Zeta-potential of SP-CDs, *S. aureus*, *E. coli* and MREC (with or without SP-CDs treatment). (**b**) *S. aureus*, *E. coli* and MREC (with or without SP-CDs treatment).

The surface of the SP-CDs contained a large number of amino groups, and the zeta potential was +31.15 mV. The zeta potentials of *S. aureus*, *E. coli* and MREC were −10.5, −14.37 and −12.25 mV, respectively (Figure 3a), which was mainly related to teichoic acid in the cell wall of the Gram-negative bacteria and lipid and lipolysaccharide molecules in the outer membrane of the Gram-negative bacteria. The zeta potentials of *S. aureus*, *E. coli* and MREC were +21.11, +11.99 and +14.5 mV after SP-CD treatment. Membrane surface potential plays an important role in maintaining metabolic activity, cell growth and other cellular functions [25]. The high positive charge was conducive to changing the bacterial membrane potential through electrostatic interaction with the negative charge on the bacterial surface, which subsequently changes the permeability of the cell surface and leads to cell death.

#### 3.3.2. FTIR Spectra of Bacterial Cells

The wavelengths of *S. aureus* at 3675, 3281, 2971, 2901, 1644, 1529, 1451, 1394, 1229, 1078, 1006, 1051 and 879 cm^−1^ corresponded to the characteristic peaks of carboxyl and amine groups, hydroxyl in polysaccharide molecules, protein and fatty acids on the bacterial cell surface (Figure 3b). In *E. coli* and MREC, these wavelengths were basically the same, at 3675, 3281, 2971, 2900, 1647, 1539, 1450, 1393, 1241, 1077, 1046 and 879 cm^−1^, respectively. The whole spectral region was basically divided into five feature segments (I–V region). In the 3700–3200 cm^−1^ wavenumber range (region I), the strong bands had N−H and O−H tensile vibration characteristics. Thus, the absorption bands of *S. aureus* at 3675 and 3281 cm^−1^ corresponded to the amine groups and hydroxyl groups. The 3200–2700 cm^−1^ wavenumber range (region II) was dominated by C−H vibrations of fatty acids in the bacterial membrane. In this region, *E. coli* showed two characteristic peaks of C−H asymmetry and C−H symmetry at 2900 and 2971 cm^−1^. After SP-CDs treatment, the spectra of the three strains all changed significantly, the peak intensity decreased or disappeared, the number of fatty acids decreased significantly, and the cell membrane of *E. coli* changed from an ordered state to a disordered state. The amide I and amide II bands of proteins and peptides fall mainly in region III (1800–1500 cm^−1^), occurring at 1539 and 1643 cm^−1^ in *E. coli* and 1529 and 1644 cm^−1^ in *S. aureus*. After SP-CDs treatment, the peak intensity of region III decreased, indicating that the change in protein structure may be caused by cell membrane lysis. Region IV (1500–1200 cm^−1^) was controlled by the vibration spectra of proteins, fatty acids and phosphate-containing compounds. Three characteristic peaks were observed in this region near 1451, 1394 and 1229 cm^−1^. The former corresponded to the CH_2_ bending vibration of the lipid or protein, while the latter was associated with the asymmetric stretching pattern of the PO_2_^−^ groups of proteins or nucleic acids. It can be clearly seen that after SP-CDs treatment, the bacterial membrane phospholipids were destroyed. In the last zone (region V, 1200–800 cm^−1^), *E. coli* showed 1078, 106, 1051 and 879 cm^−1^ peaks, and *S. aureus* showed 1077, 106 and 879 cm^−1^ peaks, corresponding to the (C−O−C) stretching vibration of the glycosideric bond of the bacterial membrane. Among them, *S. aureus* disappeared at 1078 cm^−1^ after SP-CDs treatment, which may be a sign of cell membrane carbohydrate destruction. The results show that the chemical bonds and composition of the bacterial cell surface changed greatly, leading to cell damage.

#### 3.3.3. TEM and SEM Used to Observe Morphological Changes

**Figure 4:**
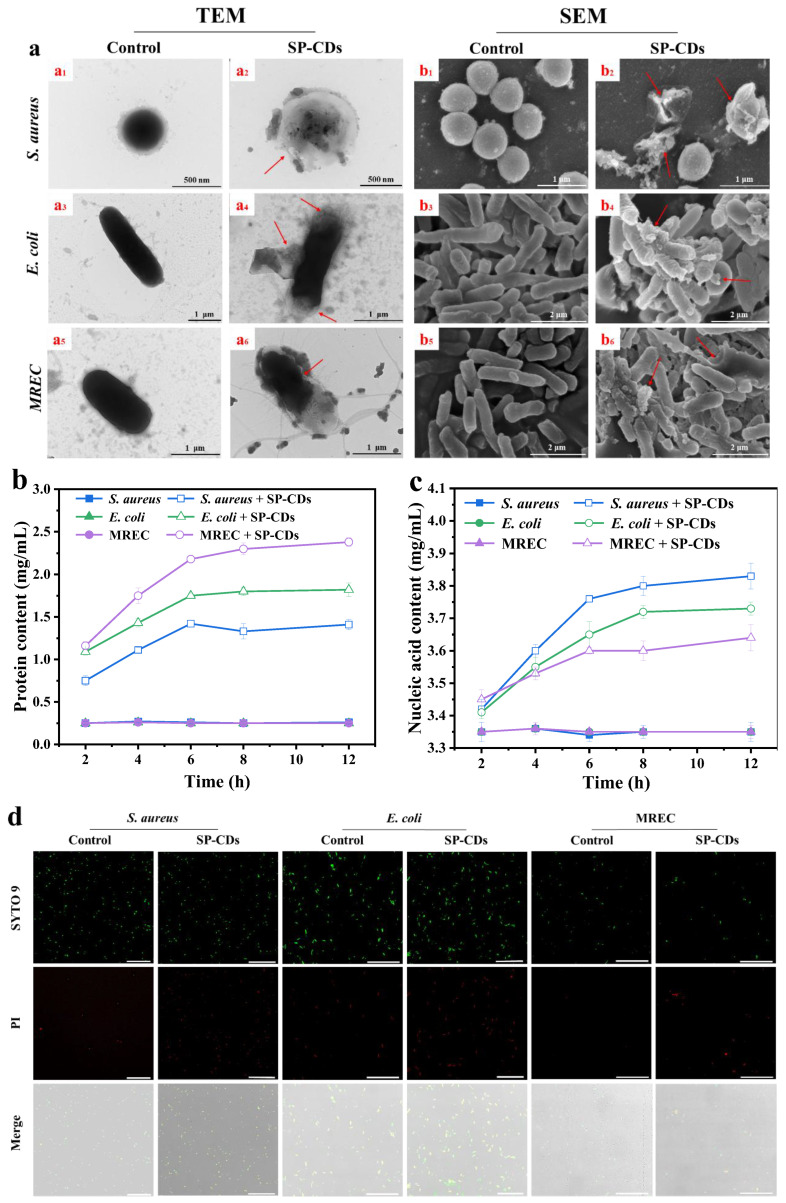
Antibacterial mechanism of SP-CDs. (**a**) TEM and SEM images of *S. aureus* (**a_1_**,**a_2_**,**b_1_**,**b_2_**), *E. coli* (**a_3_**,**a_4_**,**b_3_**,**b_4_**) and MREC (**a_5_**,**a_6_**,**b_5_**,**b_6_**) without treatment (**a_1_**,**a_3_**,**a_5_**,**b_1_**,**b_3_**,**b_5_**) or with treatment with SP-CDs (**a_2_**,**a_4_**,**a_6_**,**b_2_**,**b_4_**,**b_6_**), the arrow was added to show the disruption of membranes. Effect of SP-CDs on the (**b**) extracellular protein content and the (**c**) extracellular nucleic acid content of *S. aureus*, *E. coli* and MREC. (**d**) CLSM images of *S. aureus*, *E. coli* and MREC were stained using SYTO 9/PI dual fluorescence staining after different treatments. All bacteria alive and dead were stained using SYTO 9 showing green fluorescent intensity. Only dead bacteria can be stained by PI, emitting red fluorescent intensity. Scale bars are 50 μm. For SYTO9: λex = 488 nm; for PI: λex = 552 nm.

The TEM images of untreated bacteria preserved the clear morphology of intact and smooth membranes (Figure 4a(a_1_,a_3_,a_5_)). After being treated with SP-CDs, the cell walls and membranes of the bacteria became irregular and the cells disintegrated accompanied by intracellular leakage (Figure 4a(a_2_,a_4_,a_6_)). In addition, the untreated cells also showed clear edges and smooth walls (Figure 4a(b_1_,b_3_,b_5_)). However, many membrane defects on the surface of bacterial cells and the leakage of cell contents were found after 2 h of SP-CDs incubation (Figure 4a(b_2_,b_4_,b_6_)). These results indicate that the adsorption of SP-CDs caused physical or mechanical damage to the bacterial cell wall or outer membrane, which led to the disruption of the overall physiological function of the bacteria, or even the leakage of cytoplasmic components, ultimately leading to the death of the bacteria [30]. 

#### 3.3.4. Leak of Cell Contents

In addition, the relative amounts of bacterial extracellular proteins and extracellular nucleic acids were determined to assess whether SP-CDs inhibit bacterial growth by disrupting cell membrane integrity. The concentration of extracellular soluble proteins was significantly increased in the three strains of SP-CDs-treated bacteria compared with the control (Figure 4b). After 10 h of treatment, the protein contents of the SP-CDs-treated *S. aureus*, *E. coli* and MREC bacterial suspensions were 9.5, 7.3 and 5.6 times higher than that of the control, respectively. 

Bacterial soluble proteins are secreted proteins and enzymes involved in metabolism during the growth of bacteria. When the membrane structure of microorganisms is destroyed, a large amount of intracellular proteins leaks out, affecting the normal life activities of bacteria [26]. As shown in Figure 4c, the nucleic acid content of *S. aureus*, *E. coli* and MREC bacterial suspensions in the control group did not change significantly, while the nucleic acid content was significantly higher in the SP-CDs treatment than the control group. The above results indicate that SP-CDs can disrupt the bacteria membrane structure, leading to the leakage of a large amount of intracellular proteins and nucleic acid substances and inhibiting the growth and reproduction of bacteria.

#### 3.3.5. CLSM Used to Observe Membrane Permeability

As shown in Figure 4d, SP-CDs treatment induced a large number of dead bacteria to emit red fluorescence compared with the control group, further confirming the effective bactericidal ability of SP-CDs. The SP-CDs firstly destroy the bacterial membrane, thus acting as a bactericidal agent. The DNA of all live and dead bacteria can be stained using SYTO 9, while PI can only stain bacteria with damaged cell membranes because it cannot permeate into bacteria with intact cell membranes [38]. A large number of bacteria were stained using PI in the SP-CDs treatment group, indicating that SP-CDs had the ability to disrupt bacterial membranes.

### 3.4. Antibacterial Mechanism of SP-CDs

**Figure 5:**
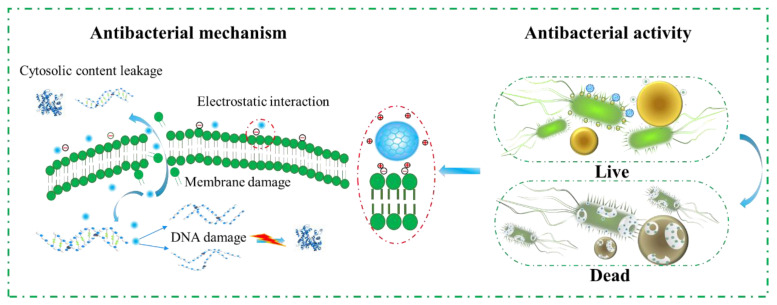
Antibacterial mechanism of the SP-CDs.

In conclusion, the antibacterial mechanism of SP-CDs was mainly attributed to damage of the bacterial cell membrane (Figure 5). The surface-capping properties of CDs, especially regarding the surface charge, is another key factor affecting their antibacterial properties. This antibacterial enhancement may be due to the positive charge of the surface coating of CDs prompting strong electrostatic interactions between the CDs and the negatively charged bacterial membrane, which further damages the integrity of the cell membrane. Positively charged SP-CDs interact with the negatively charged bacterial cell membrane through an electrostatic interaction, tightly adhering and accumulating on the bacterial surface, inducing the neutralization of surface charge and the instability of the cell membrane, increasing the membrane permeability [39]. The size of carbon quantum dots is one of the important factors that determines their interactions with cells [40]. The smaller the size of carbon quantum dots, the higher their surface-to-volume ratio, which allows the CDs to better interact with bacteria. Silver nanoparticles with a diameter of 1–10 nm have the best bactericidal effect because they preferentially interact directly with bacterial membranes [41]. Therefore, reducing the size of CDs may be an effective way to achieve a high antibacterial effect. The FTIR spectra results show that the cell membrane was changed from order to disorder and the chemical components such as fatty acids, proteins and carbohydrates in the cell membrane were disintegrated after treatment with SP-CDs. In addition, the ability of carbon quantum dots to disrupt the cell membranes was also reflected in the leakage of bacterial contents, such as nucleic acids and proteins. 

### 3.5. Antibiofilm Activity of SP-CDs

**Figure 6:**
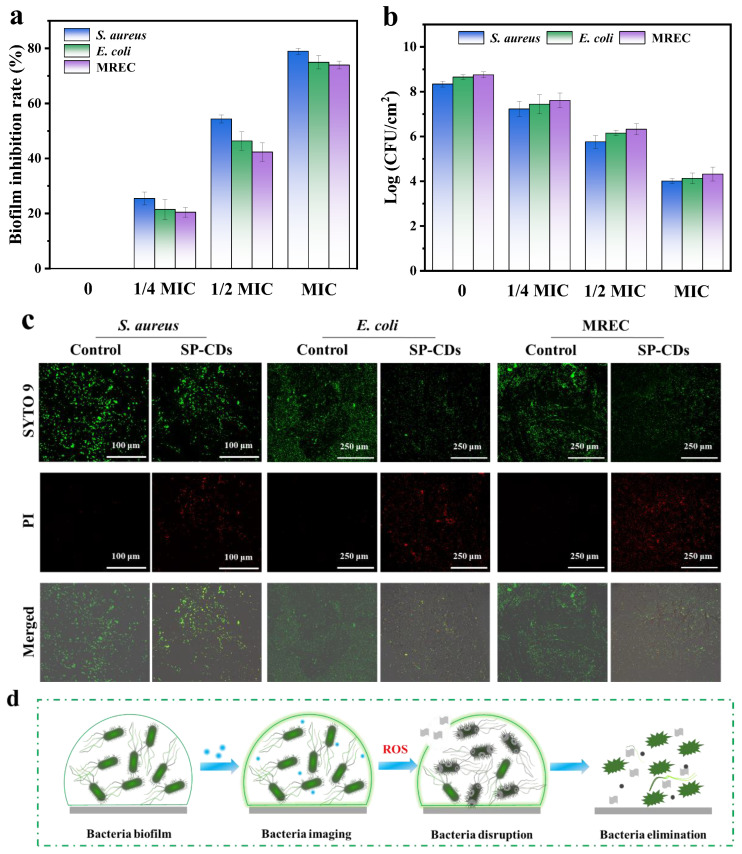
Antibiofilm activity of SP-CDs. (**a**) Biofilm inhibition rate of SP-CDs against *S. aureus*, *E. coli* and MREC. (**b**) Quantitative analysis of the relative survival rates of living *S. aureus*, *E. coli* and MREC. (**c**) CLSM images of *S. aureus*, *E. coli* and MREC were stained via SYTO 9/PI dual fluorescence staining after different treatments. All bacteria alive and dead were stained via SYTO 9 showing green fluorescent intensity. Only dead bacteria can be stained via PI, emitting red fluorescent intensity. For SYTO9: λex = 488 nm; for PI: λex = 552 nm. The scale bars of *S. aureus* are 100 μm, and the scale bars of *E. coli* and MREC are 250 μm. (**d**) Schematic diagram for SP-CDs regarding bacterial biofilm elimination.

Bacteria develop a dense biofilm that protects them from antibiotics. Therefore, after clarifying the antibacterial activity of SP-CDs, the anti-biofilm ability of SP-CDs was explored. The inhibition ability of SP-CDs against *S. aureus*, *E. coli* and MREC was determined via crystal violet staining. As shown in Figure 6a, SP-CDs treatment significantly inhibited the biofilm formation of the three bacteria, and the biofilm inhibition rate increased with increasing SP-CDs concentration. In the treatment of *S. aureus*, *E. coli* and MREC with 1 MIC SP-CDs, the biofilm inhibition rates reached 78.95%, 74.95% and 73.95%, respectively.

In addition, the plate method was used to determine the number of viable bacteria in the biofilm. The results show that SP-CDs caused dose-dependent inhibition of viable bacteria, and *S. aureus* was more sensitive to SP-CDs than *E. coli* and MREC. After dealing with the 1 MIC SP-CDs, the number of living bacteria of *S. aureus*, *E. coli* and MREC reduced from 8.34, 8.65 and 8.75 log CFU/cm^2^ to 4.01, 4.13 and 4.32 log CFU/cm^2^, respectively (Figure 6b).

The CLSM was used to study the biofilm inhibition effect of SP-CDs on *S. aureus*, *E. coli* and MREC. As shown in Figure 6c, a large area of biofilm was formed in the control group and its distribution was relatively uniform. After SP-CDs treatment, the intensity of green fluorescence decreased significantly, and a lot of red fluorescence appeared. Looser and interrupted cell clusters were observed after SP-CDs treatment. This indicated that SP-CDs can effectively inhibit bacterial attachment and reduce the formation of biofilms. The results show that SP-CDs had similar scavenging ability and inhibitory activity on biofilms. SP-CDs destroyed bacterial cell membranes by interacting with bacteria and produced more effective anti-biofilm activity by binding to DNA after entering bacteria. Therefore, SP-CDs had a higher binding rate and affinity for bacterial cell membranes, and through the natural antibacterial binding activity of SP-CDs, highly toxic reactive oxygen species (ROS) may be produced, resulting in more effective antibiofilm activity (Figure 6d).

### 3.6. Resistance Development of SP-CDs

**Figure 7:**
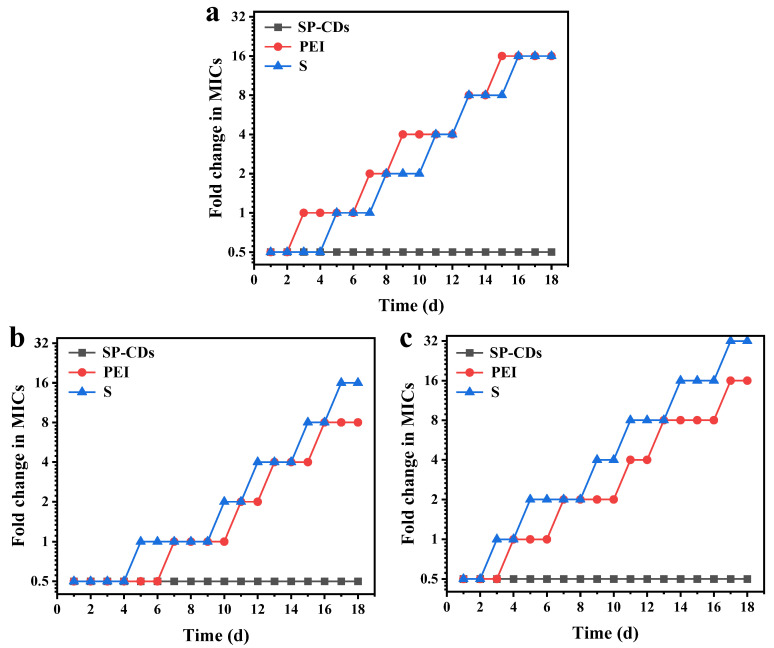
Bacterial resistance development. Resistance development of (**a**) *S. aureus*, (**b**) *E. coli* and (**c**) MREC treatment with S, PEI and SP-CDs.

The resistance development of three strains against spermidine, polyethyleneimine and SP-CDs was first assessed, and the results are shown in Figure 7. The three bacteria were cultured consecutively with SP-CDs at the level of sub-MIC for 18 d. During the treatment of 18 serial passages, the SP-CDs showed a retained antibacterial effect against the three selected bacteria without detectable resistance development (Figure 7a–c). In contrast, treatment with spermidine resulted in resistance in all three strains, with the MIC increased significantly at the 18th day; it was up to 16-, 16- and 32-fold as compared with the original MIC. In addition, the MIC of polyethyleneimine-treated strains increased by 16, 8 and 16 times. The absence of detectable antimicrobial properties in the evolution of AMR suggests that SP-CDs may be a promising antimicrobial strategy.

## 4. Conclusions

In summary, carbon quantum dots (SP-CDs) were synthesized from PEI and spermidine through a simple hydrothermal reaction, and their potential application as an antibacterial agent was explored. SP-CDs had good fluorescence performance with a large number of NH_2_ groups on their surface. In addition, SP-CDs were small in size and had a high surface positive charge, which provided high antibacterial activity against *S. aureus*, *E. coli* and multi-drug-resistant *E. coli*. The positively charged SP-CDs neutralize the surface charge of bacteria, causing damage to cell membranes, promoting the leakage of contents, and altering cell morphology and integrity. In addition, SP-CDs significantly inhibited the biofilms of the three strains. SP-CDs can effectively avoid bacterial resistance. This study provides a potential antibacterial method and provides a basis for subsequent application in the food industry.

## Data Availability

Data is contained within the article.
